# Employment status of AFROHUN-Uganda one health alumni, and facilitators and barriers to application of the one health approach: a tracer study

**DOI:** 10.1186/s12913-022-08537-7

**Published:** 2022-09-27

**Authors:** Tonny Ssekamatte, Richard K. Mugambe, Aisha Nalugya, John Bosco Isunju, Patrick Kalibala, Angella Musewa, Winnie Bikaako, Milly Nattimba, Arnold Tigaiza, Doreen Nakalembe, Jimmy Osuret, Solomon T. Wafula, Samuel Okech, Esther Buregyeya, Fatima Tsiouris, Susan Michaels-Strasser, John David Kabasa, William Bazeyo

**Affiliations:** 1 Department of Disease Control and Environmental Health, School of Public Health, Kampala, College of Health Sciences, Makerere University, P. O Box, 7072 Kampala, Uganda; 2 Africa One Health University Network, Plot 20B Kawalya Kagwa Cl, Kololo, Kampala, Uganda; 3 Makerere University College of Veterinary Medicine, Animal Resources and Biosecurity, Kampala, Uganda; 4 Columbia University, Columbia University Mailman School of Public Health, New York, NY USA

**Keywords:** Employment status, One health, Global health, Tracer study, Career development

## Abstract

**Background:**

The One Health (OH) approach integrates multiple competencies in the prevention and control of disease outbreaks. Through a range of OH competence-based activities, the Africa One Health University Network (AFROHUN) built the capacity of selected students at Makerere University and Mbarara University of Science and Technology. This study applied the Systems Theoretical Framework (STF) of career development to establish the employment status of AFROHUN-Uganda alumni, and the facilitators and barriers to application of the OH approach in their organisations.

**Methods:**

We conducted an embedded mixed-methods study among a random sample of 182 AFROHUN-Uganda alumni of the 2013–2018 cohorts. For quantitative data, descriptive statistics were computed using Stata 14.0 statistical software. A total of 12 in-depth interviews were conducted, and NVivo 12 Pro was used to organise data during thematic analysis.

**Results:**

While the majority, 87.4% were or got employed after participating in the AFROHUN Uganda capacity building programme, 68.1% were employed at the time of the survey, 57.7% had worked with their current employer for at least a year, and 39% held managerial positions. The facilitators of applying the OH approach into employing organisations included being knowledgeable about OH, the presence of a multidisciplinary workforce, the nature of activities implemented, and existing partnerships and collaborations between organisations. The barriers to the application of the OH approach included limited funding, a negative attitude towards working with people from other disciplines, and limited knowledge of the One Health approach.

**Conclusion:**

Notably, more than two-thirds of the OH alumni were employed, and more than a third held managerial position. While these findings portray a fairly good absorption rate of the OH alumni into the workforce, they also highlight the facilitators of application of the OH approach that need to be promoted as well as the barriers that need to be addressed if the application of the OH approach is to be improved within the workforce.

**Supplementary Information:**

The online version contains supplementary material available at 10.1186/s12913-022-08537-7.

## Background

The increase in global health challenges such as COVID-19 showcased the critical need for a competent one health (OH) workforce [1, 2]. Global health challenges require not only a workforce with sound technical skills and competences, but also, with the ability to work across sectors and disciplines [2, 3]. The OH approach is a proven panacea for solving global health challenges [4], however, its operationalization remains a challenge across sectors and disciplines [5–7]. The lack of effective coordination mechanisms, commitment by the different stakeholders, including government entities and other employers, has hampered the development of the OH workforce [8]. Lack of relevant policies to support institutionalization and decision-making processes, conflicting priorities between the different stakeholders, limited budgets, poor governance and leadership, and variability in sub-cultures within sectors and disciplines also remains a challenge to the operationalisation of OH [9–12].

The 2019 Berlin principles on OH recommend investment in education and raising awareness for global citizenship and holistic planetary health approaches, enhancement of capacity for cross-sectoral and trans-disciplinary health surveillance, clear and timely information-sharing to improve coordination mechanisms, participatory and collaborative relationships among stakeholders, and development of institutions that are aware of interactions at the animal, human and environment nexus [1]. Fulfilment of the Berlin principles on OH was envisioned to transform the current workforce and enable the global community to respond to global health challenges [1].

To date, academic institutions, governments, funding bodies, and networks such as the Southeast Asia One Health University Network (SEAOHUN), Malaysia One Health University Network (MyOHUN), Vietnam One Health University Network (VOHUN), Afya Bora Consortium Fellowship in Global Health Leadership, and the Africa One Health University Network (AFROHUN) (formerly known as One Health Central and Eastern Africa (OHCEA)) have immensely invested in the development of the OH workforce [2, 13–20]. These networks undertake pre-and in-service trainings with the goal of building a workforce that can apply a OH approach to predict, detect and respond to global health challenges [5, 21–23].

Given the role of the OH approach in solving global health challenges, academic institutions and OH-oriented networks continue to develop curricula that aim to empower pre-and in-service professionals with OH-related knowledge, skills and competencies [24–27]. With funding from the United States Agency for International Development (USAID), AFROHUN, which is a network of 27 institutions of veterinary and public health in the East, Central and West African region is currently championing the development of the OH workforce in ten African countries, including Uganda, Kenya, Rwanda, the Democratic Republic of Congo (DRC), Ethiopia, Tanzania, Cameroon, Liberia, and Senegal. Through AFROHUN, the Makerere University School of Public Health (MakSPH) and the College of Veterinary Medicine, Animal Resources and Bio-security (COVAB) jointly with other colleges at Makerere University designed and implemented a competence-based program aimed at developing the one health workforce in Uganda [4, 5, 28, 29]. The program aims to build OH competencies of interdisciplinary and cross-sectoral teams through field experiential learning, graduate fellowships, undergraduate innovations, OH student clubs, OH resident training, MSc. Scholarships, and 16 OH theoretical modules/courses [4, 5, 28, 29].

The theoretical courses and field experiential learning offered to all the AFROHUN-Uganda alumni aimed to build the pre-and in-service human resources and bolster the workforce for more effective detection, prevention and response to global health threats such as COVID-19 [4]. It was expected that the acquisition of OH skills and competencies among the pre-and in-service professionals would increase their absorption in the job market. Consequently, these would apply the acquired knowledge, skills and competencies to solve local and global health challenges, and to bridge the human resource gaps that are evident at local and international levels [30, 31]. However, there are insights that different institutions lack adequate finances to employ multi-disciplinary teams to facilitate the application of the OH approach, and data to demonstrate how investment in animal or environmental health can benefit public health, especially when the health threat is not immediate [32, 33]. A lack of policies further compromises the ability of organisations to employ individuals trained in OH [33]. Despite these circumstances, there is still limited evidence of the employment status of the OH alumni, and the relevance, barriers and facilitators of applying the OH approach at workplaces employing the alumni.

**Fig. 1:**
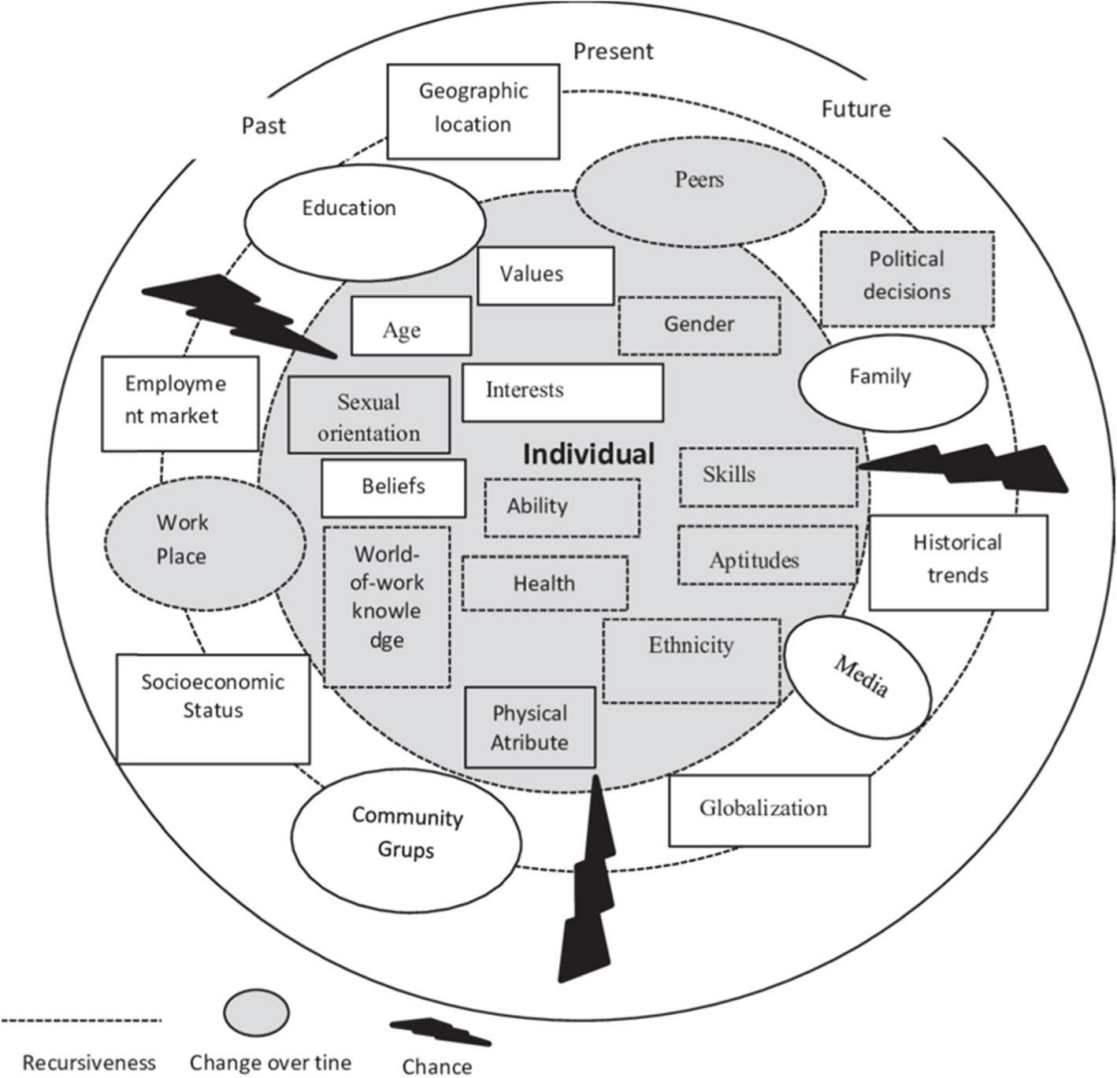
Systems theoretical framework [34]

In this study, the Systems Theoretical Framework (STF) of career development (Fig. 1) was used to establish the employment status of AFROHUN-Uganda OH alumni, and to understand the relevance, facilitators and barriers to the application of the OH approach by alumni in their current employment. Based on this framework, individual characteristics such as gender, values, sexual orientation, ability, interests, skills, age, the world of work knowledge, physical attributes, aptitudes, education level, education background, ethnicity, personality, beliefs, disability, health status, and values are critical for someone to get employed, and consequently apply the knowledge acquired through the theoretical and experiential learning [34]. The STF suggests that the employment status and consequently application of OH knowledge, skills and competencies can also be affected by the social system including peers, friends and family, education institutions, the media, workplace conditions, and the environmental/societal system (for example political decisions) [34]. The STF was chosen as the appropriate model since employment is often the goal of career development services [35].

## Materials and methods

### Study setting

**Fig. 2:**
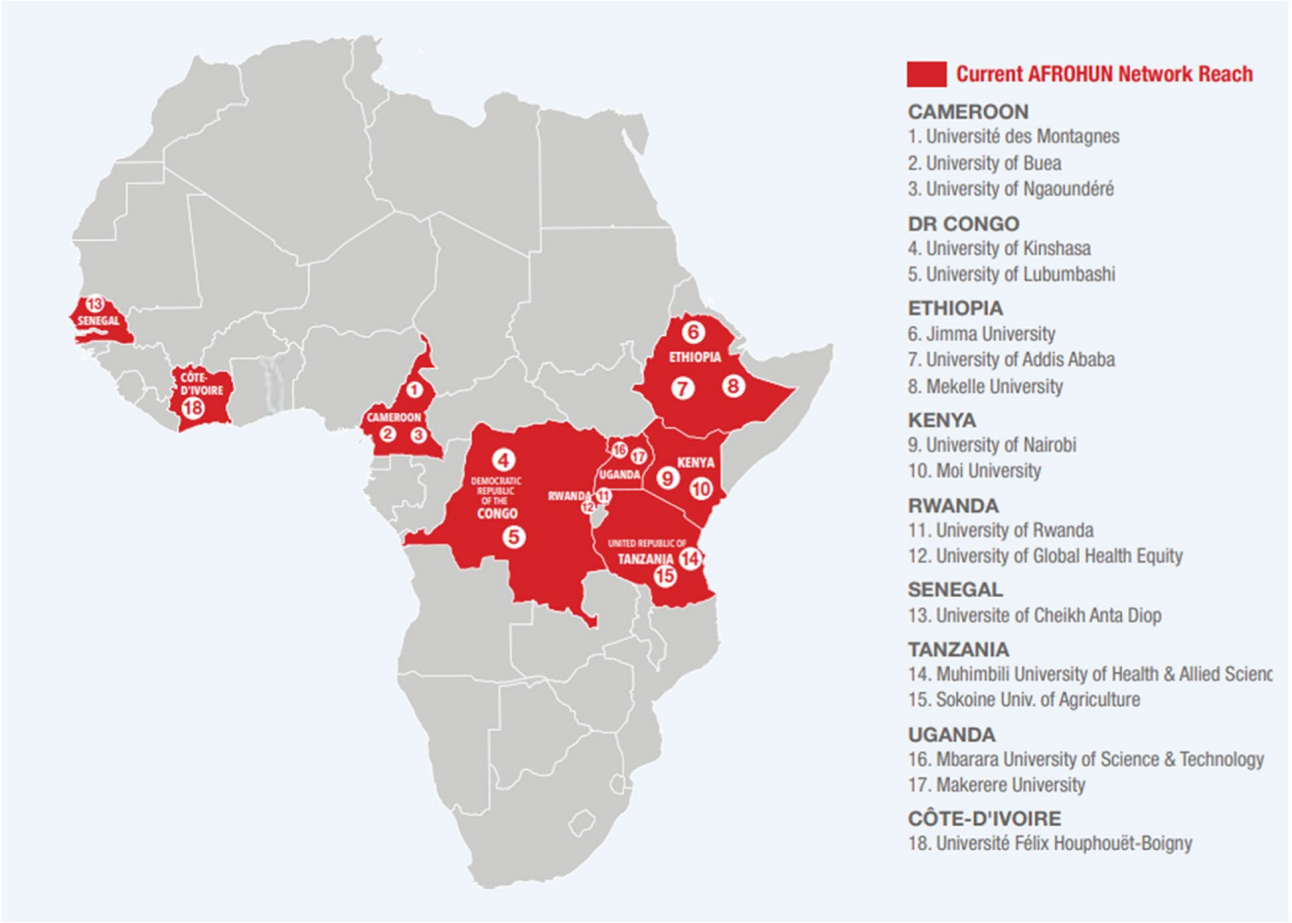
Distribution of AFROHUN member countries and institutions [14]

The current study was conducted among the AFROHUN-Uganda alumni from the 2013–2018 cohorts of Makerere University and Mbarara University of Science and Technology. AFROHUN is a network of institutions of higher education consisting of 27 Schools of public health, veterinary medicine, pathobiology, environmental sciences, and medicine (Fig. 2). These schools are in 19 universities in ten countries in East, Central and West African regions (Uganda, Cameroon, Democratic Republic of Congo, Ethiopia, Kenya, Rwanda, Senegal, Côte D’Ivoire, Liberia, and Tanzania) [14]. The network is comprised of The University of Ngaoundéré and University of Buea (Cameroon), Universite des Montagnes, University Félix Houphoet Boigny (UFHB) (Côte D’Ivoire), the University of Lubumbashi and University of Kinshasa (DRC), Jimma University, Addis Ababa University and Mekelle University (Ethiopia), Moi University and University of Nairobi (Kenya), Université Cheikh Anta Diop (Senegal), Muhimbili University of Health and Allied Sciences and Sokoine University of Agriculture (Tanzania), the University of Rwanda and University of Global Health Equity (Rwanda), and Makerere University and Mbarara University of Science and Technology (Uganda) [14]. Since its inception in 2010, AFROHUN has played a key role in building the capacity of the OH workforce. By 2021, a total of 4555 students and 1432 faculty had been trained across the different countries [14]. All the AFROHUN alumni in the network countries undertake theoretical courses on 1) collaboration and partnership, 2) OH management, 3) culture and ethics, 4) behaviour change, 5) communication, 6) leadership, 7) OH outbreak investigation, 8) OH systems thinking, 9) risk analysis, 10) policy and advocacy, 11) OH research, 12) OH principles and concepts, 13) infectious disease management, 14) gender and OH, 15) epidemiology, and 16) ecosystem health [14, 28].

### Study design and data collection procedures

This was an embedded mixed-methods study. An embedded mixed methods design is useful when a single data set is not sufficient to answer the research questions [36], as was the case in the current study. During the implementation of the study we first used quantitative data collection methods to establish employment status, and then applied qualitative methods to explore the relevance, barriers and facilitators of the OH approach in organisations that employed the alumni. These data were partly used to explain the employment status of the respondents.

### Sample size estimation and sampling

The sample size was estimated using the Kish Leslie formula for cross-sectional studies [37]

$${\displaystyle \begin{array}{c}X=\Big[\frac{\left[\left({z}^2\ast p\ast \Big(1-p\right)\right]}{\left[\Big({ME}^2\right]}\\ {}X=\Big[\frac{\left[\left({1.96}^2\ast 05\ast \Big(1-0.5\right)\right]}{\left[\Big({0.05}^2\right]}\end{array}}$$

The estimated conservative proportion of AFROHUN Uganda alumni who are employed was assumed at 50%. A 95% level of confidence, an error rate of 0.05 and a Z score of 1.96 corresponding to the two 95% confidence interval (CI) was used in the calculation, which yielded a sample size of 384. Given that the calculated sample size (384) was higher than the total population of alumni trained (308) by over 5%, we applied Daniel’s sample size correction formula.

N = effective sample size.

n = Total population of alumni.

$${\displaystyle \begin{array}{c}N=\frac{X}{1+X/n}\\ {}N=\frac{384}{1+384/308}=170\end{array}}$$

Considering a non-response of 10%, the total sample size was 189 alumni.

The detailed sampling methodology has been described in our earlier publication [4]. Briefly, respondents were selected using simple random sampling. A random sample was generated from a list of the AFROHUN Uganda alumni using the Ms. EXCEL random number generator function (RAND formula: =RAND ()). The contact details (such as email addresses and phone numbers) of selected respondents were obtained from the AFROHUN-Uganda country office and or their former colleges. We also requested the participants to share contact details of other participants, selected from the same cohort as the participant especially if reliable contacts could not be obtained from the AFROHUN-Uganda country office. To understand the facilitators and barriers to the application of the OH approach among the alumni, a total of 12 in-depth interviews were conducted. Interviewees were purposively selected based on their demographic characteristics and employment status.

### Data collection methods and tools

A structured questionnaire was used to obtain quantitative data from the respondents. The structured questionnaire was preloaded on the KoboCollect data collection application (App),which was preinstalled on smart phones and tablets. Depending on the availability of the respondents, the research Assistants emailed or “WhatsApped” the respondents with the link giving them access to the electronic questionnaire, used phone interviews, and/or online interviews (Skype and Zoom). However, face to face interviews were considered as the core approach to data collection in this study. These approaches were adopted given that they had been successful in previous tracer studies; most notably those conducted by the Uganda National Council of Higher Education (NCHE) [38] and L Macatangay [39]. In addition, participants were also asked which data collection mode suits them the most so it is used. This was so because majority of the participants did not have sufficient time for face to face interviews. We modified a structured questionnaire which was used by the NCHE to conduct a tracer study of the 2005 graduates from five universities and four colleges in Uganda, to obtain quantitative data from the selected alumni [38]. The questionnaire was designed in such a way that it captured information on the respondents’ background characteristics and their employment status (supplementary file 1). The questionnaire was reviewed for content and construct validity by a team of one health experts in the AFROHUN network (https://afrohun.org/). In order to ensure face validity [4], the questionnaire was also pretested among 10 alumni of the 2019 cohort. Pre-test of the questionnaire provided an opportunity for the core research team to improve clarity of the questions. The qualitative component of the study used an in-depth interview guide to capture data on the facilitators and barriers to the application of the OH approach at their workplaces. The in-depth interview guide elicited information on what makes it possible for the employee’s organisation to apply a multidisciplinary approach in solving day to day programmatic challenges, whether the employee’s organisational culture supports a multi-disciplinary approach to solving day to day challenges, steps taken by the employee’s organisation to promote the one health approach, and what makes it difficult to promote a multidisciplinary approach to solving health challenges in the employee’s workplace. The questions contained in the in-depth interview guide were developed after a critical review of literature on barriers and facilitators of effective implementation of the one health approach [27, 40–42]. After the development of the interview guide, it was pretested among 4 AFROHUN alumni of the 2019 cohort to ensure that the questions were clear and well-understood prior to the main data collection phase. Both the questionnaire and the interview guide were evaluated for face and content validity by a team of OH experts within AFROHUN and other partner institutions. At the time of data collection, each qualitative interview lasted about 30–45 minutes while the quantitative interview lasted 20 minutes.

### Study variables, data management, and analysis

#### Quantitative data

Employment status was categorized as a binary outcome. A respondent was classified as being employed if they had a job, volunteer ship or an internship opportunity where he/she received salary, wage or honorarium or any other benefits for their services. Data were collected and entered using mobile phones and tablets pre-loaded with Kobo Collect mobile application. Data were downloaded in Ms. Excel format and cleaned. Data were analysed using Stata 14.0 statistical software (Statacorp Texas, USA). Descriptive analyses such as frequencies, proportions, and means were performed for the employment status of the alumni and their socio-demographic characteristics. Cross-tabulations were done to compare the employment status of the alumni by sex. Sex/gender-based discrimination negatively impacts employment opportunities [43–45], and consequently the OH workforce.

#### Qualitative data

Data were collected on the facilitators and barriers to the application of the OH approach by the alumni in their current workplaces. All qualitative interviews were conducted in English. Interviews were audio-recorded to reduce recall bias by the researcher. The audio files were then transcribed verbatim. Thematic content analysis guided the identification of themes, i.e., patterns in the data that are important or interesting, and use these themes to address the study objectives. All transcripts were coded using the NVivo 12 Pro, after which they were categorised into themes. Themes were then developed and summarized using a data master sheet. Data generated from the quantitative component were triangulated with that from the qualitative (IDIs) to give more meaning to the study findings (Supplementary file 2). Reporting of qualitative findings has been informed by the Consolidated criteria for reporting qualitative studies (COREQ) (supplementary file 3). A force-field analysis framework was also used to rate the forces that facilitated or hindered the application of the one health approach [46]. During the rating, each respondent was asked to score on a 5-point Likert how important the different facilitators and barriers impacted the application of the OH approach at their workplaces. During the scoring, facilitators and barriers that were scored 5 had the most influence on the application of the OH approach. At decision-making level, increasing or maintaining the scores for the facilitators, and reducing the scores for the barriers was expected to enhance the application of the OH approach at workplaces.

#### Quality control and assurance

Access to data was restricted to only the principal investigators (TS, JBI, RKM, KP and AN) and the data manager (ND) who had the security key to the Kobo Collect server, where the data was sent during data collection. Research assistants (RAs) were recruited from a pool of experienced RAs. The RAs were trained on the research protocol and ethical issues pertaining to the study to ensure quality data collection. The PIs only recruited RAs who are well conversant with English which was used by the study participants. The data entry screen was designed with skips and restrictions to ensure quality data entry.

## Results

### Background characteristics of the respondents

A total of 182 respondents were interviewed, representing a response rate of 96.3%. More than half, 58.8% (107/182) were males. The mean age of the respondents was 28.7 ± 4.6, the median age was 27 while the modular age was 26 years. The mean age at the award of the undergraduate degree was 24.6 years ±2.9. About a quarter, 24.2% (44/182) of the respondents had attained a Master’s degree, while almost three quarters, 73.6% (134/182) had attained a bachelor’s degree as their highest level of academic qualification (Table 1).

**Table 1:** Background characteristics of the respondents

Variable	Attribute	Frequency (***N*** = 182)	Percentage (%)
Sex	Male	107	58.8
	Female	75	41.2
Age in years	Below 30	136	74.7
	30 and above	46	25.3
Year of attendance of any AFROHUN-Uganda capacity building program	2012–2015	37	20.3
	2016–2018	145	79.7
Nature of OH activities that the alumni participated in^a^	One health field attachment	163	89.6
	One health students’ club	8	4.4
	Master of Veterinary Public Health and Management	3	1.6
	Got a scholarship	2	1.1
	Fellowship	22	12.1
	Out Break Investigations	20	11.0
	OH residency	2	1.1
	Innovations	11	6.0
Had any other academic qualification before the award of the most recent qualification	Yes	22	12.1
	No	160	87.9
Highest level of academic qualification	Bachelors	134	73.6
	Masters	44	24.2
	Post Graduate Diploma	4	2.2

^a^Multiple response variable

### Employment status of the AFROHUN-Uganda 2013–2018 OH alumni

The majority, 87.4% (159/182) got employed after participation in the AFROHUN-Uganda capacity building programme. On average, the alumni had ever worked for two employers. More than two-thirds, 68.0% (124/159) had been employed at the time of the survey, and 77.7% (96/124) had worked with their current employer for at least a year. About 68.7% (46/75) of the females compared to 84.8% (78/107) of the males had been employed at the time of the survey. About 16.4% (26/159) had jobs that were not related to their fields of study. Close to a third, 22.4.0% (15/75) of the males while 12.0% (11/107) of the females did not have jobs related to their field of study. Of these, 65.4% (17/26) had jobs that were not related to their fields of study due to limited opportunities in their career, 27.0% (7/26) due to personal reasons, diversification and need to seek an alternative career, 7.6% (2/26) chose a different career path due to lack of career progression and poor working conditions. More than two-thirds, 66.7% (106/159) had worked for at least two employers since participation in the AFROHUN-Uganda capacity building program (Table 2).

**Table 2:** Employment status of the AFROHUN-Uganda 2013–2018 OH alumni stratified by sex

Variable	Category	Overall	Sex	
			Female	Male
Got employed after participation in the AFROHUN-Uganda capacity building programme	Yes	159 (87.4)	67 (81.3)	92 (86.0)
	No	23 (12.6)	8 (10.7)	15 (14.0)
Currently employed (*n* = 159)	Yes	124 (68.0)	46 (68.7)	78 (84.8)
	No	35 (22.0)	21 (31.3)	14 (15.2)
The duration between getting a job and engagement with AFROHUN-Uganda (*n* = 159)	Less than a year	106 (66.6)	42 (62.7)	64 (60.4)
	At least a year	30 (18.9)	17 (25.4)	13 (14.1)
	Not applicable	23 (14.5)	8 (11.9)	15 (16.3)
Still employed by first employer (*n* = 124)	Yes	53 (42.7)	20 (43.5)	33 (42.3)
	No	71 (57.3)	26 (56.5)	45 (57.7)
Duration of working with current employer/ being self-employed (*n* = 124)	Less than a year	28 (22.6)	14 (30.4)	14 (18.0)
	At least a year	96 (77.4)	32 (69.6)	64 (82.1)
Terms of employment (*n* = 124)	Permanent/Full-time	54 (43.6)	18 (39.1)	36 (46.2)
	Contract	56 (45.2)	24 (52.2)	32 (41.0)
	Part-time	4 (3.2)	1 (2.2)	3 (3.9)
	Temporary	3 (2.4)	2 (4.4)	1 (1.3)
	Self-employed	4 (3.2)	0 (0.0)	4 (5.1)
	Volunteer	2 (1.6)	0 (0.0)	2 (2.6)
	Other	1 (0.8)	1 (2.2)	0 (0.0)
Sector where the respondent works (*n* = 155)	Agriculture	18 (11.6)	3 (4.6)	15 (16.7)
	Water and Sanitation	15 (9.7)	8 (12.3)	7 (7.8)
	Research	33 (21.3)	10 (15.4)	23 (25.6)
	ICT	6 (3.9)	3 (4.6)	3 (3.3)
	Trade/ business/ Entrepreneurship	17 (11.0)	7 (10.8)	10 (11.1)
	Veterinary/ wildlife sector	24 (15.5)	7 (10.8)	17 (18.9)
	Health sector	46 (29.7)	20 (30.8)	26 (28.9)
	Tertiary education	11 (7.1)	2 (3.1)	9 (10.0)
	Other sectors	36 (23.2)	19 (29.2)	17 (18.9)
Mechanism for getting the job (*n* = 159)	Through an application	107 (67.3)	46 (68.7)	61 (67.3)
	Through a friend	12 (7.6)	6 (9.0)	6 (6.5)
	Self employed	2 (1.3)	0 (0.0)	2 (2.2)
	Through recommendation	34 (21.4)	14 (20.9)	20 (21.7)
	Other	4 (2.5)	1 (1.5)	3 (3.3)
Number of employers worked for since participation in AFROHUN-Uganda capacity building program (*n* = 159)	One employer	53 (33.3)	21 (32.3)	27 (33.3)
	At least two	106 (66.7)	44 (67.7)	54 (66.7)
Current job related to field of study (*n* = 159)	Yes	133 (83.7)	52 (77.6)	81 (88.0)
	No	26 (16.4)	15 (22.4)	11 (12.0)

^a^Multiple responses. Other sectors include financial management, social work and public administration, and construction/civil engineering and security

### Reasons for not being in active employment

**Fig. 3:**
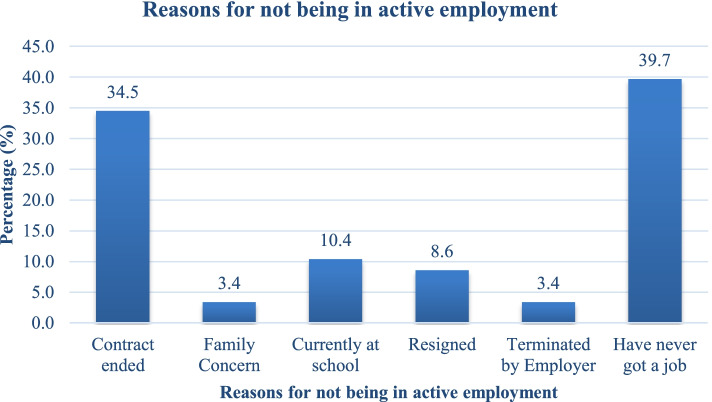
Reasons for not being in active employment

Out of those who were currently unemployed, 34.5% (20/58) had had their contracts ended by their employers, 10.4% (6/58) were currently in school while 3.4% (2/58) had had their contracts terminated (Fig. 3).

### Relationship between AFROHUN-Uganda one health training activities and employment status

Only 18.7% (34/182) were employed by the time they participated in AFROHUN-Uganda One health activities. More than half, 65.9% (120/182) got a new job or promotion after completing the AFROHUN-Uganda OH activities. Slightly more than a third, 39% (71/182) were employed in managerial positions, and 56.7% (89/157) had always been gainfully employed since participation in AFROHUN-Uganda OH activities. Only 34.2% (25/75) of the females compared to 43.0% (46/107) of the males were employed in managerial positions. About 50.8% (33/75) of the females while 60.9% (56/107) of the males had always been gainfully employed since participating in AFROHUN-Uganda OH activities. Almost a third, 31.8% (50/157), to a very high extent, applied the acquired one health activities in their most recent job (Table 3).

**Table 3:** Relationship between AFROHUN-Uganda One Health training activities and employment

Variable	Category	Overall N (%)	Sex	
			Female (***n*** = 75)	Male (***n*** = 107)
Employed by the time you participated in AFROHUN-Uganda OH activities	Yes	34 (18.7)	13 (17.3)	21 (19.6)
	No	148 (81.3)	62 (82.7)	86 (80.4)
Got a new job or promotion since completing the AFROHUN-Uganda OH activities	Yes	120 (65.9)	47 (64.4)	73 (68.2)
	No	62 (34.1)	26 (35.6)	34 (31.8)
Employed in a managerial position in your organisation	Yes	71 (39.0)	25 (34.2)	46 (43.0)
	No	111 (61.0)	48 (65.8)	61 (57.0)
Been gainfully employed since participating in AFROHUN-Uganda OH activities (*n* = 157)	Never	8 (5.1)	4 (6.2)	4 (4.4)
	Yes, for some time	60 (38.2)	28 (43.1)	32 (34.8)
	Yes, always	89 (56.7)	33 (50.8)	56 (60.9)
The extent of use of the acquired knowledge and skills during AFROHUN-Uganda OH activities in your most recent job (*n* = 157)	Very high extent	50 (31.8)	20 (30.8)	30 (32.6)
	High extent	46 (29.3)	28 (43.1)	28 (30.4)
	Some extent	56 (35.7)	15 (23.1)	31 (33.7)
	Limited extent	4 (2.6)	2 (3.1)	2 (2.2)
	Not at all	1 (0.6)	0 (0.0)	1 (1.1)
Characterization of the relationship between AFROHUN-Uganda OH activities and most recent job (*n* = 159)	AFROHUN-Uganda OH activities are by far the best in relation to my current job	92 (57.9)	46 (68.7)	46 (50.0)
	Other fields of study could prepare me for this job as well	38 (23.9)	14 (20.9)	24 (26.1)
	Another field of study would have been more useful for this job	5 (3.1)	0 (0.0)	5 (5.4)
	The field of study does not matter very much for this job	16 (10.1)	4 (6.0)	12 (13.0)
	Other	8 (5.0)	3 (4.5)	5 (5.4)

### Relevance, facilitators and barriers to the application of the OH approach

**Fig. 4:**
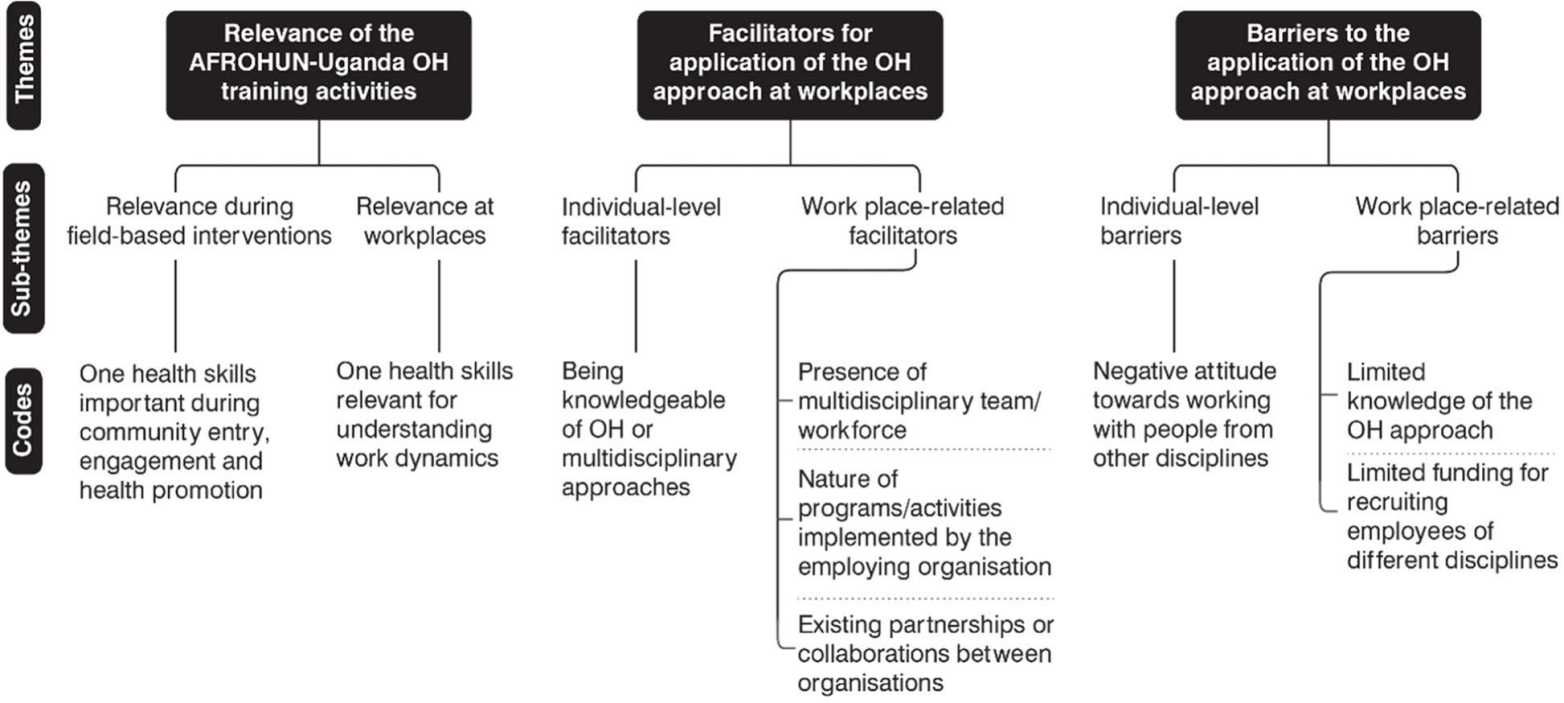
Relevance, facilitators and barriers to the application of the OH approach at workplaces of the AFROHUN-Uganda 2013–2018 alumni

Through the qualitative component, respondents revealed the relevance, facilitators and barriers to the application of the OH approach at workplaces. The OH approach was relevant for community entry, engagement and health promotion and understanding workplace dynamics. At an individual level, knowledge of OH or multidisciplinary approaches was vital for the application of the approach. The workplace-related facilitators included the presence of a multidisciplinary workforce, the nature of programs/activities implemented by the employer, and existing partnerships or collaborations between employers. At individual level, a negative attitude towards working with people from other disciplines and limited knowledge of the OH approach hindered its application. Limited funding for recruiting employees of different disciplines hindered the application of the OH approach (Fig. 4).

### Relevance of the AFROHUN-Uganda OH training activities

#### Relevance of OH during the implementation of field-based interventions

##### One health skills important during community entry, engagement and health promotion

The AFROHUN-Uganda training activities provided skills that were required at the workplaces of the alumni. The respondents mentioned that skills learnt through field-based interventions were required at the alumni workplaces. Such skills helped some alumni to mainstream WASH activities in the prevention and control of infectious disease threats.

*“The AFROHUN-Uganda activities provided me skills which are relevant to my recent job. Through the community-based interventions, I obtained in community entry and engagement and health promotion, which are critical at my workplace”* (Female participant).

“The knowledge and skills obtained from the AFROHUN-Uganda OH training activities are relevant especially when I use them to integrate the component of WASH (water, sanitation and hygiene) in my fieldwork. I always undertake WASH mainstreaming in my activities since it is key in the prevention of many infectious diseases and other waterborne related infections. I learnt and observed this while participating in the AFROHUN field attachment. I currently apply one health knowledge during the sensitisation of the community members. Thus, I find AFROHUN’s work relevant” (Male participant).

#### Relevance of OH at workplaces

##### One health skills relevant for understanding work dynamics

The combination of theories and practical during OH activities enabled the alumni to understand work dynamics. Through interaction with the different stakeholders, the alumni acquired knowledge and skills in team building and teamwork. Besides, participation in the OH activities helped the alumni to develop critical thinking skills. Individuals who participated in outbreak investigations pointed out that such activities built their capacity in obtaining information from people and innovate solutions for detecting infectious disease threats. These activities also changed the attitude of the alumni toward working with individuals from different disciplines and sectors.

*“The combination of theories and practical helped us understand workplace dynamics. The involvement of all the stakeholders such as government leaders made us understand how to relate with people and how to implement activities the right way”* (Female participant).

*“The outbreak investigation helped me to learn how to get information from people, I became aware of infectious diseases and outbreak investigation. It also helped me to quickly design a quick tool that can be used for capturing information on a possible outbreak”* (29-year-old participant).

*“Working in interdisciplinary teams and the field placement taught us how to approach and solve community challenges sustainably”* (28 years old participant).

*“The practical exposure given to us during the one health field placement was invaluable. It accorded us an opportunity to propose our own solutions while in the field. This was vital in enhancing critical thinking & problem-solving skills”* (30 years old participant).

*“AFROHUN Uganda uses a multi-disciplinary approach that helps all disciplines get involved in its work. This helped to build our teamwork skill set”* (27-year-old participant).

### Facilitators of application of the OH approach at workplaces

#### Individual-level facilitators of application of the OH approach

##### Being knowledgeable on one health or multidisciplinary approaches

This study revealed that the application of the multidisciplinary approach in tackling workplace or community challenges was facilitated by prior training in the OH approach or other multidisciplinary approaches. Employees that had undergone the OH training with AFROHUN felt that they could embrace working with colleagues from any discipline, thus fostering the application of the OH approach.

*“Personally, because I am knowledgeable of a multi-disciplinary approach that I learnt from OHCEA, I can embrace working with any discipline”.* (Female participant).

#### Work place-related facilitators of application of the OH approach

##### Multidisciplinary workforce

Application of the OH approach was possible due to the presence of multidisciplinary teams in some of the organisations employing the AFROHUN Uganda OH alumni. Some organisations also employed interns from disciplines such as environmental health, public health, and adult and community education. These interns complemented the efforts of the full-time staff at the organisations employing the AFROHUN-Uganda OH alumni, thereby making the application of the one health field attachment a reality. Besides, some organisations held multidisciplinary meetings where views were taken from all staff irrespective of their discipline. It is such meetings that facilitated the application of the OH approach to work challenges.

“*… first of all, we have a multidisciplinary team. We also have interns who usually come from different disciplines. For instance, we have interns with a background in public health, adult education, and environmental health. When these interns come on board, they try to compliment the staff here. The interns and the team we have here embrace the multidisciplinary approach.”* (27-year-old participant).

*“The organization organises meetings where everyone is free to discuss any challenges faced, and also suggest possible solutions to addressing those challenges.”* (30-year-old participant).

##### Nature of programs/activities implemented by the employers/organisation

Application of the OH approach at workplaces depended on the nature of activities implemented by the different organizations. Organizations that focused on cross-cutting issues were more likely to adopt a multidisciplinary approach compared to those that did not. The approach was seen as critical in addressing the different community challenges. Workplaces that focused on only one issue such as those dealing with legal matters were less likely to use the one health or multidisciplinary approach. The respondents from such workplaces, however, did not downplay the role the OH approach would play in achieving the goals of the organisations. It was for instance mentioned that some of the GBV victims they represent in court would benefit from the approach since they also need psychosocial support.

*“Our program includes health education on sexually transmitted diseases including HIV/AIDS. So, as we do HIV testing during the outreach, we also sensitise the community on how to wear condoms and how to construct tippy taps. It requires multiple disciplines to conduct these activities. So that also facilitates the application of the one health approach in the work we do.”* (35 year old).

*“We need each other in some way or the other. As a lawyer, I cannot solve every person’s problems (respondent laughs). One health is necessary especially when it comes to handling legal aspects of a woman who has been raped or a girl who has been defiled. I think it’s essential that they (woman who has been raped and the child who has been defiled) get these other services! Of psychological support, you get it! As a lawyer my point of view is different from that of a doctor, you get it! Yeah, they need this support and all that; I think it would be nice to partner with other disciplines, you get it!”* (Female participant).

##### Existing partnerships or collaborations between organisations

Partnerships or collaborations between the different organisations was key in the application of the OH approach. These organisations provided an opportunity for employees with varying disciplines to interact with their counterparts from other organisations, thereby promoting the OH approach.

*“We are working closely with people of different disciplines from KCCA and the ministry of water which have staff of different disciplines. We are working with monitoring and Evaluation specialists, sanitation specialists, environmental engineers and water quality assistants. This provides us with an opportunity to work as a multidisciplinary team”* (28-year-old participant).

*“When you go to the field, as Reproductive Health Uganda is conducting vaccinations and malaria check-ups, you set up a legal desk to hand legal matters, which I think is more enriching.”* (38-year-old participant).

### Barriers to the application of the OH approach at workplaces

#### Individual-level barriers to the application of the OH approach at workplaces

##### Negative attitude towards working with people from other disciplines

The application of the OH approach was also hindered by the negative attitude of some employees who felt that they were more superior to their counterparts. These felt that they understood their programs best and were not willing to appreciate ideas from their counterparts.

*“You know some people feel they understand their programs best. So, when you bring on board someone else from another team, for example, from reproductive health to help out with WASH, they may feel they feel that this person is talkative, active and overshadowing them. Certain people always feel they know more than others because they are of a particular discipline, you know! They do not appreciate ideas from other people just because they are not from their disciplines, they feel they know more because they studied what they are implementing.”* (35-year-old participant).

#### Workplace related barriers to the application of the OH at workplaces

##### Limited funding

The application of the OH approach into organisations employing the OH alumni was hindered by the limited funding to bring onboard employees of different disciplines. Respondents pointed out that available funds would only cater for one or two individuals which limit multi-disciplinarily collaboration.

*“You may find that as much as we want to bring so many people on board to tackle a particular problem, the funds available may only allow for one or two individuals. The people who run that program can only pay one or two people. So, resources are one of the challenges.”* (Female participant).

##### Limited knowledge of the OH approach

Some respondents pointed out that the application of the OH approach in the operations of their organisations was limited by a lack of awareness of the multi-disciplinary approach. As a result, many organisations recruited staff with a particular discipline, thereby promoting working in silos.

*“Many organizations are “green” (ignorant) about the multi-disciplinary approach.*

*So, even at recruitment, you find organizations wanting to employ individuals of only a particular discipline. Therefore, it is a lack of awareness, people are not aware!”* (Male, participant).

##### Force field analysis of facilitators and barriers to the application of the OH approach at workplaces

**Fig. 5:**
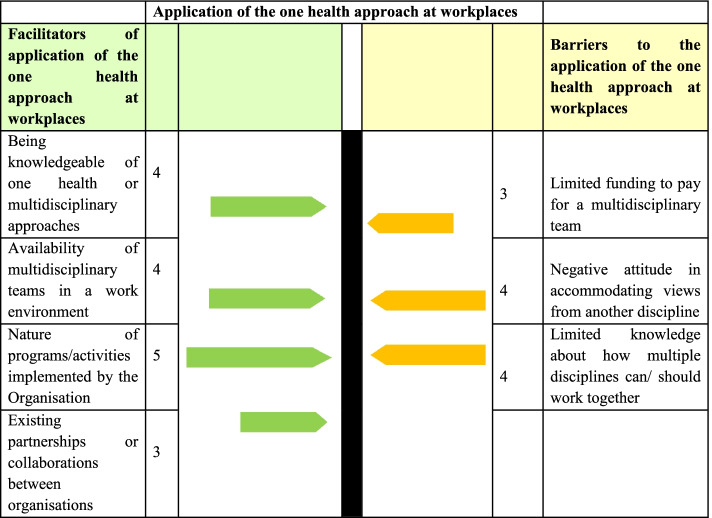
Force field analysis of facilitators and barriers to the application of the OH approach at workplaces among the 2013–2018 AFROHUN alumni

The scores for the facilitators were 5 for the nature of activities/programs, 4 for being knowledgeable of OH or multidisciplinary approaches, and availability of multidisciplinary teams in a work environment. The scores for barriers were 4 for limited knowledge about how multiple disciplines can/ should work together and negative attitude in accommodating views from another discipline and 3 for limited funding to pay for a multidisciplinary team (Fig. 5).

## Discussion

The current study established the employment status of the AFROHUN-Uganda OH alumni, and the relevance, facilitators and barriers to the application of the OH approach in their current employment. More than two-thirds of the alumni were in active employment and nearly three quarters had jobs related to their fields of study. The facilitators included; being knowledgeable of OH or multidisciplinary approaches, presence of a multidisciplinary workforce, nature of programs/activities implemented by the organisation, and existing partnerships or collaborations between organisations. The barriers included limited funding, a negative attitude towards working with people from other disciplines, and limited knowledge of the OH approach.

More than two-thirds of the alumni in the current study were in active employment. The employment rate among the alumni is relatively high and could be explained by the discipline-specific opportunities that exist in Uganda. As indicated in the current study, experience garnered through participation in OH was relevant in the current employment of the alumni. It was evident that the alumni obtained skills such as team building and teamwork, which were relevant for understanding workplace dynamics. Participating in multidisciplinary global health training programmes is often associated with an improvement in leadership, analytic and communication and problem-solving competences [4, 47], which are often desired by employers. Therefore, it could be these skills that attracted their employers, thus explaining the high employment rates. The employment rate reported in our study is consistent with that reported among the master of global health alumni [48] and lower than that reported among the Afya Bora Consortium Fellowship in Global Health Leadership (Afya bora) alumni [20]. For the alumni in the current study, engaging in OH activities may have increased their chances of employment [49]. Global health training programmes such as those implemented by AFROHUN incorporate experiential learning, which increases their chances of employment [50–53]. The difference in the proportion of alumni in active employment reported in our study and the former could be the nature of the trainees. The AFROHUN-Uganda training involves pre and in-service trainees while the Afya Bora capacity building programme involves in-service participants with solid work experience. Moreover, all the Afya bora alumni in a previous study were engaged in leadership positions in government departments and nongovernmental organizations in their countries [20].

Nearly a third of the alumni were not in active employment, even amidst the pandemic where their knowledge and skills are required. The current COVID-19 pandemic and other emerging and re-emerging infectious disease threats provide an opportunity for the application of the OH approach. However, this was not the case, since a third of the alumni were unemployed. The unemployment rate among a third of the alumni is not surprising given the high unemployment rates that characterise many countries in the global south, and Uganda in particular [54–56]. Furthermore, low-income countries have limited and fragmented funding for global health initiatives [57] and demand for healthcare workers [58], which compromises the ability of the different employers to bring on board multi-disciplinary teams. Our study, therefore, suggests the need for OH health training institutions to emphasize entrepreneur skills which can enable students to start their own jobs and enterprises. Stakeholders advancing the OH approach should, for example, train students in research and grant writing in order for them not to be job seekers rather job creators.

Our study revealed that nearly three-quarters of the alumni had jobs related to their fields of study. However, a considerable proportion chose a different career path due to lack of career progression, limited job opportunities in their career, poor working conditions, personal reasons, career diversification and the need to seek an alternative career. The fact that some alumni opt to undertake jobs not related to their careers is not surprising. The change in career path among the alumni may have resulted from poor pay at their workplaces, as reported among other professionals [59–62]. The high proportion of alumni who had jobs related to their fields of study can be explained by the nature of the alumni enrolled for the AFROHUN training programs. Majority of the alumni have a background in animal or human health sciences such as veterinary medicine, environmental health and medicine which are highly marketable despite the worrying economic situations. None the less, OH competencies are applicable in various disciplines and settings so could still be useful in jobs outside fields of training.

Our study also revealed that more than two-thirds of the alumni got a new job or promotion after completing the AFROHUN-Uganda OH activities. New employment opportunities obtained by the alumni may be attributed to the skills that the alumni obtained through participating in OH activities such as the one health institute (OHI), graduate placements, and scholarships. Through the OHI, the alumni obtain skills in project management, leadership, infectious diseases management, communication, collaboration, epidemiology, behavioural change among others, which are desired in the job market [63–65]. This is further explained by the fact that the majority of the alumni to some extent used the knowledge and skills acquired during the AFROHUN-Uganda One health activities at their most recent job and the fact that AFROHUN-Uganda One health activities were by far the best in relation to more than half of the alumni’s current jobs.

Besides the employment status, the study also sought to understand what facilitates application of the OH approach in the current employment of the alumni. We found out that being knowledgeable of OH or multidisciplinary approaches was critical for its application in the current employment. Knowledge of OH and its benefits may have changed the attitude of the alumni toward collaboration with individuals from other disciplines, thus discouraging working in discipline-specific silos. Knowledge has previously been shown to influence attitude [66]. In addition, the OH alumni in these organisations may have shared their knowledge and skills, which would ultimately stimulate a multidisciplinary and collaborative approach to work.

The OH approach was possible in some organisations given the nature of the activities they engage in. Some organisations have programs that span various disciplines, thus making a multi-disciplinary and multi-sectoral workforce feasible. Some organisations for instance engaged in the construction of sanitation infrastructure, meaning that they already had engineers, behavioural scientists/sociologists, and environmental health scientists on board. At the same time such organisations engaged in disease surveillance thereby making the involvement of medical doctors, nurses and other clinical cadres inevitable. Such organisations may have joint meetings where all professionals are invited to contribute to the broader project/program or organizational goals. On the contrary, organisations that only focused on one line of business or thematic areas such as computing and legal matters provided limited avenues for the application of the OH approach.

Existing partnerships or collaborations between the different organisations were critical in fostering the OH approach in such organisations. Partnerships between the different organisations provided an opportunity for the formation of multisectoral and multidisciplinary teams. Some organisations for example worked with local authorities, ministries, departments and agencies, which usually have different professionals and staff of varying disciplines (such as monitoring and evaluation specialists, sanitation specialists, environmental engineers, water quality specialists and medical doctors among others). Working with such organisations helped them to team up with professionals from different backgrounds, thereby enabling them to utilize the OH approach in achieving their project objectives and goals.

Limited funding impeded the use of the one health approach in organisations employing the AFROHUN Uganda OH alumni [42, 67–69]. The current study revealed that organisations employing one health alumni did not have adequate funds to facilitate the application of the OH approach into their programs. It is important to note that the application of the OH approach requires bringing on board individuals from diverse backgrounds to solve common health or environmental challenges. Therefore, a lack of funds to remunerate individuals from diverse disciplines crippled the implementation of the OH approach. The limited funding that characterizes organisations employing OH alumni should worry stakeholders concerned with the prevention of health challenges at the environment-animal-human interface since attainment of optima animal, human and environmental health is largely dependent on a multi-disciplinary and multi-sectoral approach. The limited funding that characterizes organisations that employ OH alumni should stimulate alternative approaches for fostering the OH approach. Health-related organisations working on antimicrobial stewardship can for example work hand in hand with animal-health related organisations as well as environment/biodiversity related organisations to improve anti-microbial stewardship. This would enable the attainment of optimal human-animal-environmental health without necessarily increasing budgets.

A negative attitude towards working with people from other disciplines was a barrier to the application of the OH approach into the organisations employing the OH alumni. Whereas some efforts have been made to sensitise the different stakeholders at the animal-human-environment interface about the role of the one health approach in averting health challenges, individual attitudes relating to teamwork remain unfavourable. The current study reveals that many stakeholders at the environment-animal-human interface still work in silos. The negative attitude towards working with individuals from other disciplines may have resulted from job insecurity and lack of employment opportunities in the country. This may tempt individuals from one discipline not to join individuals of other disciplines in solving OH challenges. This study also revealed that individuals from some disciplines opted not to work with others with the belief that individuals from other cadres did not understand the kind of work they do. This study illustrates a level of mistrust and insecurity among the different cadres. Therefore, there is a need to break the negative attitude among employers in order to achieve the objectives of the one health approach. This can be achieved through in-service trainings targeting individuals in organisations whose mission is to advance health and wellbeing.

Limited knowledge of the OH approach in organisations employing the OH alumni hindered the utilisation of the approach. This study revealed that, due to limited knowledge of the approach, many organisations recruited individuals from just one discipline, which hindered a collaborative, multisectoral and transdisciplinary approach. The limited knowledge of the one health approach in organisations employing the OH approach could have resulted from the limited in-service trainings for individuals who are already employed. Nonetheless, the existence of OH alumni in some of these organisations should be capitalized on. The alumni can be useful in the transfer of one health-related knowledge and competencies to their less competent colleagues. Besides, there is a need to conduct OH trainings to bridge the knowledge and competence gap in organisations working towards optimal animal, environmental and human health.

### Strengths and limitations

This study may have been the first to establish the employment status of one health alumni, and the barriers and facilitators of the application of the one health approach in organisations employing the one health alumni. It used an appropriate sample size, established from those who had participated in the one health program hosted by AFROHUN-Uganda. However, our study had some limitations. Using virtual platforms such as Zoom for data collection was characterised by poor internet connectivity which affected the quality of audio recordings, and consequently the transcripts which were used for analysis. This meant that some information may have been lost during the course of recording. As a countermeasure, each interview was conducted by two researchers, among whom, was a note taker, whose role was to summarise all the responses given by the respondent. The researchers requested the respondents to elaborate on the responses that were not clear during the course of the interviews, thus reducing the risk of loss of vital information. Some of the respondents in our study were engaged in the COVID-19 response which made it difficult for them to provide ample time for the interviews. This meant that the conversations between the research team and the respondents were shorter than would otherwise have been, consequently affecting data saturation. Failure to reach data saturation meant that our qualitative results and conclusions may be short of some key themes and subthemes [70]. Finally, our study was conducted only among the AFROHUN alumni in Uganda, and thus our findings cannot be generalized to the other 9 countries in the network or other health training programmes due to the differences in the training modalities. A more comprehensive study encompassing alumni in the different AFROHUN network countries and partner institutions may address limitation related to generalizability of findings.

### Reflexivity

The findings of the current study should be interpreted and utilised while putting the following into perspective. All the authors listed in this paper have in one way or the other been involved in the implementation of one health activities under the AFROHUN. WB is the AFROHUN Chief Executive Officer, JDK is a co-investigator for the one health workforce project, EB is a former one health focal person for the Makerere University School of Public Health (MakSPH), while AM is the country manager for AFROHUN Uganda. SO was in 2016 the one health field attachment focal person for the Makerere University College of Veterinary Medicine, Animal Resources and Biosecurity. TS, who conceptualised and led the study is a former one health field attachment focal person for MakSPH and he is currently the thematic lead for workforce assessment and tracking. TS is currently leading the development of a strategy to define a one health worker in Uganda and engages in periodic supervision and monitoring of the AFROHUN one health alumni in Uganda. AN, AT and DN are currently supporting the development of a strategy to define a one health worker in Uganda.

## Conclusions

Our study revealed that more than two-thirds of the AFROHUN-Uganda OH alumni were in active employment, and nearly three quarters had jobs related to their fields of study. The facilitators of application of the OH approach into employing organisations included; being knowledgeable of one health or multidisciplinary approaches, presence of a multidisciplinary team/workforce, nature of programs/activities implemented by the Organisation, and existing partnerships or collaborations between organisations. The barriers included limited funding, a negative attitude towards working with people from other disciplines, and limited knowledge of the OH approach. The current study reveals the need to develop job creators rather than seekers, and to innovatively integrate the OH approach in organisations through fostering collaborations among organisations, rather than increasing the number of staffs amidst the current financial crisis. Based on our force field analysis, there is an urgent need to enhance and/or maintain knowledge of OH at workplaces and existing partnerships or collaborations between organisations. There is also a need to address barriers such as limited funding, low knowledge of the OH approach and a negative attitude toward OH in organizations.

## Supplementary Information

**Additional file 1.**

**Additional file 2.**

**Additional file 3.**

## Data Availability

The datasets analysed during the current study are available from the corresponding author upon reasonable request.

## Abbreviations

AFROHUN: Africa One Health University Network
OH: One Health
OHCEA: One Health Central and Eastern Africa
OHI: One Health Institute
STF: Systems Theory Framework

## References

[1] Gruetzmacher K, Karesh WB, Amuasi JH, Arshad A, Farlow A, Gabrysch S, Jetzkowitz J, Lieberman S, Palmer C, Winkler AS (2021). The Berlin principles on one health–bridging global health and conservation. Sci Total Environ.

[2] One Health Workforce: Developing a global workforce to prevent, detect, and respond to infectious disease threats. https://www.usaid.gov/sites/default/files/documents/1864/OHW_Overview_Handout_2016-ct-508-1.pdf.

[3] WHO (2022). National workforce capacity to implement the essential public health functions including a focus on emergency preparedness and response: roadmap for aligning WHO and partner contributions.

[4] Ssekamatte T, Isunju JB, Nalugya A, Mugambe RK, Kalibala P, Musewa A, Bikaako W, Nattimba M, Tigaiza A, Nakalembe D (2022). Using the Kolb’s experiential learning cycle to explore the extent of application of one health competencies to solving global health challenges; a tracer study among AFROHUN-Uganda alumni. Glob Health.

[5] Atusingwize E, Ndejjo R, Tumukunde G, Buregyeya E, Nsamba P, Tuhebwe D, Kato CD, Naigaga I, Musoke D, Kabasa JD (2020). Application of one health approach in training at Makerere University: experiences from the one health workforce project in Uganda. One Health Outlook.

[6] Lee K, Brumme ZL (2013). Operationalizing the one health approach: the global governance challenges. Health Policy Plan.

[7] Gibbs EPJ (2014). The evolution of one health: a decade of progress and challenges for the future. Vet Rec.

[8] Asaaga F, Young J, Oommen M, Chandarana R, August J, Joshi J, Chanda M, Vanak A, Srinivas P, Hoti S (2021). Operationalising the “one health” approach in India: facilitators of and barriers to effective cross-sector convergence for zoonoses prevention and control. BMC Public Health.

[9] Poma LD (2022). Systems’ barriers and facilitators of one health programs that address zoonotic diseases: a review of the literature. Int J Infect Dis.

[10] Abuzerr S, Zinszer K, Assan A (2021). Implementation challenges of an integrated one health surveillance system in humanitarian settings: a qualitative study in Palestine. SAGE Open Med.

[11] Muhammad-Bashir B, Halimah BA (2022). Challenges and future perspectives for the application of one health. One Health.

[12] Queenan K, Garnier J, Rosenbaum N, Buttigieg S, de Meneghi D, Holmberg M, Zinsstag J, Rüegg SR, Häsler B, Kock R (2017). Roadmap to a one health agenda 2030. CAB Rev.

[13] Streichert LC, Sepe LP, Jokelainen P, Stroud CM, Berezowski J, Del Rio Vilas VJ. Participation in One Health Networks and Involvement in the COVID-19 Pandemic Response: A Global Study. Front Public Health. 2022. 10.3389/fpubh.2022.830893. 10.3389/fpubh.2022.830893 PMC8907588 35284359

[14] Introducing and Welcoming you to Africa One Health University Network (AFROHUN). https://afrohun.org/.

[15] One Heath digest. A quarterly publication of the Africa One Health University Network. https://afrohun.org/wp-content/uploads/2021/07/AFROHUN-One-Health-Digest-Jan-March-2021.pdf.

[16] Amuguni H, Bikaako W, Naigaga I, Bazeyo W (2018). Building a framework for the design and implementation of one health curricula in east and Central Africa: OHCEAs one health training modules development process. One Health (Amsterdam, Netherlands).

[17] Häsler B, Bazeyo W, Byrne AW, Hernandez-Jover M, More SJ, Rüegg SR, Schwarzmann O, Wilson J, Yawe A (2020). Reflecting on one health in action during the COVID-19 response. Front Vet Sci.

[18] Nguyen-Viet H, Ratanakorn P, Adisasmito W, Bin Omar B, Fenwick S, Mukti A (2012). South East Asia one Health University network (SEAOHUN): a regional network for one health capacity building.

[19] Nguyen-Viet H, Lam S, Nguyen-Mai H, Trang DT, Phuong VT, Tuan NDA, Tan DQ, Thuy NT, Linh DT, Pham-Duc P (2022). Decades of emerging infectious disease, food safety, and antimicrobial resistance response in Vietnam: the role of one health. One Health.

[20] Voss J, Yasobant S, Akridge A, Tarimo E, Seloilwe E, Hausner D, et al. Gaps, Challenges, and opportunities for Global Health leadership training. Ann Glob Health. 2021;87(1). 10.5334/aogh.3219 PMC8284509 34307065

[21] Amuguni H, Bikaako W, Naigaga I, Bazeyo W. Building a framework for the design and implementation of one health curricula in east and Central Africa: OHCEAs one health training modules development process. One Health. 2019;7. 10.1016/j.onehlt.2018.08.002 PMC6288412 30569012

[22] Chatterjee P, Chauhan AS, Joseph J, Kakkar M (2017). One Health/EcoHealth capacity building programs in South and South East Asia: a mixed method rapid systematic review. Hum Resour Health.

[23] Ogunseitan O (2022). Competencies for one health workforce quality assurance: disciplinary diversity and consensus in a global eDelphi panel. Int J Infect Dis.

[24] Buregyeya E, Atusingwize E, Nsamba P, Nalwadda C, Osuret J, Kalibbala P, Nuwamanya R, Ssekamatte T, Wakabi T, Bikaako W (2020). Benefits of a community based interdisciplinary learning exposure: a qualitative study of the one health approach in teaching at Makerere University, Uganda. Research Square.

[25] Controlling Zoonotic Diseases through a One Health Approach. https://sites.globalhealth.duke.edu/dukeonehealth/one-health-training-program/.

[26] One Health Workforce - Next Generation. https://ohi.vetmed.ucdavis.edu/programs-projects/one-health-workforce-next-generation.

[27] Berrian AM, Wilkes M, Gilardi K, Smith W, Conrad PA, Crook PZ, Cullor J, Nyatanyi T, Smith MH, Kazwala R (2020). Developing a global one health workforce: the “Rx One Health Summer Institute” approach. EcoHealth.

[28] One Health Modules. https://afrohun.org/course/onehealthmodules/.

[29] Ssekamatte T, Tetui M, Kibira SP, Isunju JB, Mugambe RK, Nabiwemba E, Wafula ST, Buregyeya E, Bukenya JN (2020). Multiple sexual partnerships and associated factors among young psychoactive-substance-users in informal settlements in Kampala, Uganda. Plos One.

[30] World Health Organization (2016). Global strategy on human resources for health: workforce 2030.

[31] Michel J-P, Ecarnot F, editors. The shortage of skilled workers in Europe: its impact on geriatric medicine, vol. 11. Switzerland: Springer; 2020. p. 345–7. 10.1007/s41999-020-00323-0 PMC7176573 32328964

[32] Munyua PM, Njenga MK, Osoro EM, Onyango CO, Bitek AO, Mwatondo A, Muturi MK, Musee N, Bigogo G, Otiang E (2019). Successes and challenges of the one health approach in Kenya over the last decade. BMC Public Health.

[33] Okello AL, Bardosh K, Smith J, Welburn SC (2014). One health: past successes and future challenges in three African contexts. Plos Negl Trop Dis.

[34] Patton W, McMahon M (2006). Connecting theory and practice: the systems theory framework of career development and career counselling. Int J Adv Couns.

[35] McMahon M. Work and why we do it: a systems theory framework perspective. Career Plan Adult Dev J. 2017;33(2).

[36] Creswell JW, Plano Clark VL (2011). Choosing a mixed methods design. Design Conduct Mixed Methods Res.

[37] Kish L. Sampling organizations and groups of unequal sizes. Am Sociol Rev. 1965:564–72. 14325826

[38] NCHE (2013). Tracer study of 2005 graduates from five universities and four colleges.

[39] Macatangay L (2013). Tracer study of BSCS graduates of lyceum of the Philippines University from 2004-2009. Acad Res Int.

[40] Buregyeya E, Atusingwize E, Nsamba P, Musoke D, Naigaga I, Kabasa JD, Amuguni H, Bazeyo W (2020). Operationalizing the one health approach in Uganda: challenges and opportunities. J Epidemiol Glob Health.

[41] Kelly TR, Machalaba C, Karesh WB, Crook PZ, Gilardi K, Nziza J, Uhart MM, Robles EA, Saylors K, Joly DO (2020). Implementing one health approaches to confront emerging and re-emerging zoonotic disease threats: lessons from PREDICT. One Health Outlook.

[42] Kayunze KA, Kambarage DM, Kiwara A, Lyamuya E, Rushton J, Kock R, Coker R (2014). Practice of one health approaches: bridges and barriers in Tanzania: proceedings. Onderstepoort J Vet Res.

[43] Nye-Lengerman K, Narby C, Pettingell S (2017). What is the relationship between gender and employment status for individuals with IDD?. Findings from the National Core Indicators Adult Consumer Survey (Bringing Employment First to Scale, Issue No. 9).

[44] Cifre E, Vera M, Sánchez-Cardona I, De Cuyper N. Sex, gender identity, and perceived employability among Spanish employed and unemployed youngsters. Front Psychol. 2018;2467. 10.3389/fpsyg.2018.02467 PMC6292941 30581404

[45] The gender employment gap: Challenges and solutions. https://ec.europa.eu/eurostat/cros/system/files/43-2015-the_gender_employment_gap-challenges_and_solutions.pdf.

[46] Brown PR, Meyer SB (2015). Dependency, trust and choice? Examining agency and ‘forced options’ within secondary-healthcare contexts. Curr Sociol.

[47] Gachuno O, Odero T, Seloilwe E, Urassa D, Tarimo E, Nakanjako D, Sewankambo N, Atanga NS, Halle-Ekane EG, Manabe Y (2021). AFYA BORA CONSORTIUM FELLOWSHIP: a journey of success in Global Health leadership training. Afr Health Sci.

[48] Cherniak W, Nezami E, Eichbaum Q, Evert J, Doobay-Persaud A, Rudy S, et al. Employment opportunities and experiences among recent master’s-level global health graduates. Ann Global Health. 2019;85(1). 10.5334/aogh.305 PMC6634463 30873801

[49] Palazuelos D, Dhillon R, Nelson AK, Savage KP, Conover R, Katz JT, Rhatigan JJ (2018). Training toward a movement: career development insights from the first 7 years of a global health equity residency. J Grad Med Educ.

[50] Anjum S (2020). Impact of internship programs on professional and personal development of business students: a case study from Pakistan. Future Bus J.

[51] Galbraith D, Mondal S. The potential power of internships and the impact on career preparation. Res Higher Educ J. 2020;38.

[52] Jung J, Lee SJ (2017). Impact of internship on job performance among university graduates in South Korea. Int J Chin Educ.

[53] Baert BS, Neyt B, Siedler T, Tobback I, Verhaest D (2021). Student internships and employment opportunities after graduation: a field experiment. Econ Educ Rev.

[54] World Bank. Unemployment, youth total (% of total labor force ages 15-24) (modeled ILO estimate) - Uganda. 2020. [cited 2021 15-08]. Available from: https://data.worldbank.org/indicator/SL.UEM.1524.ZS?locations=UG.

[55] Pletscher M (2015). Youth unemployment in Uganda: roots of the problem and possible ways to mitigate them.

[56] Lakuma CP, Marty R, Kuteesa A (2016). Survival analysis of regional unemployment in Uganda: evidence from the Uganda National Panel Survey (UNPS). Afr Dev Rev.

[57] Gichaga A, Masis L, Chandra A, Palazuelos D, Wakaba N (2021). Mind the global community health funding gap. Glob Health.

[58] Liu JX, Goryakin Y, Maeda A, Bruckner T, Scheffler R (2017). Global Health workforce labor market projections for 2030. Hum Resour Health.

[59] Chima SC (2020). Doctor and healthcare workers strike: are they ethical or morally justifiable: another view. Curr Opin Anesthesiol.

[60] Nankwanga A, Neema S (2020). Access to health and healthcare among older persons in Uganda. Health and Care in old age in Africa.

[61] Otremba M (2012). When doctors become creditors: the detainment of impoverished patients in Uganda. Essay And Documentary Film.

[62] Akello G, Beisel U (2019). Challenges, distrust, and understanding: employing communicative action in improving Trust in a Public Medical Sector in Uganda. SAGE Open.

[63] Sonnenschein K, Ferguson J (2020). Developing professional communication skills: perceptions and reflections of domestic and international graduates. J Univ Teach Learn Pract.

[64] McKenna CJ (2019). Alumni perspectives on mission-critical communication skills for new job-market entrants. Fed Bus Discip J..

[65] Trisnaningsih S, Sutrisno S, Permatasari Y, Hendra FH, Sulistyowati E (2020). Contingency model to increase the uptake of higher education graduates in the job market. J Asian Finance Econ Bus.

[66] Ssekamatte T, Isunju JB, Zirimala PAK, Etajak S, Kamukama S, Seviiri M, Nakafeero M, Nalugya A, Tsebeni Wafula S, Atusingwize E (2021). A positive attitude among primary healthcare providers predicts better hepatitis B prevention practices: evidence from a cross-sectional survey in Wakiso district, Central Uganda. Health Psychol Behav Med.

[67] Asaaga FA, Young JC, Oommen MA, Chandarana R, August J, Joshi J, Chanda MM, Vanak AT, Srinivas PN, Hoti SL (2021). Operationalising the “One Health” approach in India: facilitators of and barriers to effective cross-sector convergence for zoonoses prevention and control. BMC Public Health.

[68] Rwego IB, Babalobi OO, Musotsi P, Nzietchueng S, Tiambo CK, Kabasa JD, Naigaga I, Kalema-Zikusoka G, Pelican K (2016). One health capacity building in sub-Saharan Africa. Infect Ecol Epidemiol.

[69] Fasina FO, Fasanmi OG, Makonnen YJ, Bebay C, Bett B, Roesel K (2021). The one health landscape in sub-Saharan African countries. One Health.

[70] Saunders B, Sim J, Kingstone T, Baker S, Waterfield J, Bartlam B, Burroughs H, Jinks C (2018). Saturation in qualitative research: exploring its conceptualization and operationalization. Qual Quant.

