# Supplementary material for: Employment status of AFROHUN-Uganda one health alumni, and facilitators and barriers to application of the one health approach: a tracer study
Source: BMC Health Serv Res. 2022 Sep 27;22:1205. doi: 10.1186/s12913-022-08537-7 (PMC9513298; doi:10.1186/s12913-022-08537-7)
Supplement: Supplementary file 1 — Additional file 1. [file 12913_2022_8537_MOESM1_ESM.docx]

# **Structured questionnaire for the one health alumni**

**Study tittle**: Situation analysis of employment status of OHCEA-Uganda 2012-2017 one health alumni, and opportunities and facilitators of integration of the one health approach into their organisations.

| This tracer study is intended for you as an AFROHUN-Uganda One Health alumna. Its purpose is to assess your employment status following your participation in AFROHUN-Uganda one health activities. You will respond to an online question or computer aided interview about some questions pertaining your employment history, and the extent of integration of the One Health approach in your organisation, if employed. | | | |
| --- | --- | --- | --- |
| **No** | **Question** | **Responses** | **Skip pattern** |
| 101 | Gender of respondent | 1. Male 2. Female |  |
| 102 | Age in complete years |  |  |
| 103 | Home district |  |  |
| 104 | In which year did you attend any AFROHUN-Uganda capacity building program? |  |  |
| 105 | In what AFROHUN-Uganda capacity building activities did you participate in? (Multiple responses allowed) | \| Activity \| Participated \| Year \| Duration of participation \| \| --- \| --- \| --- \| --- \| \| One Health field attachment \| Y/N \|  \|  \| \| One Health students’ club \|  \|  \|  \| \| Master of Veterinary Public Health and Management \|  \|  \|  \| \| Got a scholarship \|  \|  \|  \| \| Fellowship \|  \|  \|  \| \| Out Break Investigations \|  \|  \|  \| \| One Health residency \|  \|  \|  \| \| Innovations \|  \|  \|  \| | |
| 106 | Age at award of undergraduate |  |  |
| 107 | Did you have any other academic qualification prior to the award of the most recent qualification? | 1. Yes 2. No | if No, skip the next question |
| 108 | If yes, what course |  |  |
| 109 | Highest academic qualification | 1. Bachelors 2. Masters 3. 3. PhD |  |
| 110 | Year of award |  |  |
|  | **Employment status** | |  |
| 201 | Have you ever got employed since you completed the field attachment? | 1. Yes  2. No |  |
| 202 | Are you currently employed? | 1. Yes  2. No | If No, go to next question |
| 203 | How long did you take before getting a job after your engagement with AFROHUN? | ………..Months  …………..Years  Not applicable (already had a job) |  |
| 204 | Is your current employer your first one? | 1. Yes 2. No |  |
| 205 | How long have you been working for your current employer/ been self-employed? | Months……………  Years……….  (Choose either months or years) |  |
| 206 | How would you classify the setting in which you work? | 1. Rural 2. Urban 3. Abroad |  |
| 207 | Terms of employment | 1. Never Been Employed 2. Self employed 3. Permanent/Full Time 4. Part Time 5. Self-employed 6. Family Business 7. Temporary 8. Contract |  |
| 208 | Reasons for not being employed | 1. Contract ended 2. Terminated by Employer 3. Resigned 4. Family Concern 5. Sickness 6. Never had a job |  |
| 209 | Name of most current/ last employer/ organisation |  |  |
| 210 | In what sector would you classify the activities of your organization? | 1. Agriculture 2. Water and Sanitation 3. Research 4. ICT 5. Trade/ business/ Entrepreneurship 6. Veterinary/ wildlife sector 7. Health sector 8. Tertiary education 9. Other (specify) |  |
| 211 | Region of work |  |  |
| 212 | Years of paid work since the field attachment |  |  |
|  | How did you get to know about the job? | 1. News paper 2. Radio 3. Internet/ Email 4. Door to door hunting 5. Through a friend/ relative 6. Through a Friend/Relative 7. Through internship/internal advert 8. Other (specify) |  |
| 213 | How did you get the job? | 1. Applied 2. Through a friend 3. Self employed 4. Recommended 5. Taken on after being a One health intern |  |
| 214 | How many employers did you work for before the current one? |  |  |
| 215 | How long since the attachment did you take to get employed? (If you have never been employed since then, indicate 00) |  |  |
| 216 | Is your current job related to your field of study/discipline? | 1. Yes 2. No |  |
| 217 | If No, why did you change your career? | 1. Lack of career progression 2. Poor remuneration 3. Poor working conditions 4. Inappropriate Skills 5. Limited Opportunities in my career 6. Other (please specify) ................................ |  |
| 218 | Does your current job require application of One Health knowledge and skills? | 1. Yes 2. No |  |
| 219 | If Yes, what kind of skills? |  |  |
| 220 | Did you learn any employable skills from the AFROHUN-Uganda One health activities? | 1. Yes 2. No | if No, skip the next question |
| 221 | If yes, what skills did you learn? | 1. Community engagement 2. Community entry 3. Project planning and management 4. Report writing 5. Data analysis 6. Communication skills 7. Problem solving skills 8. Other (specify) |  |
| 222 | Did you use any of these skills while in search for employment? | 1. Yes 2. No | if No, skip the next question |
| 223 | If yes, what skills did you use? | 1. Community engagement 2. Community entry 3. Project planning and management 4. Report writing 5. Data analysis 6. Communication skills 7. Problem solving skills 8. Other (specify) |  |
| 224 | Did you attend an interview for any job after participating in the AFROHUN-Uganda One health activities? | 1. Yes 2. No | if No, go to question |
| 225 | If yes, did the interviewers ask if you had any of these skills? (Tick all that apply) | 1. Community engagement 2. Community entry 3. Project planning and management 4. Report writing 5. Data analysis 6. Communication skills 7. Problem solving skills 8. Other (specify) |  |
| 226 | If yes, did you qualify for the job? | 1. Yes 2. No |  |
| 227 | If self-employed, please list the skills acquired from the one health field attachment programme that you are using in your work? | 1. Community engagement 2. Community entry 3. Project planning and management 4. Report writing 5. Data analysis 6. Communication skills 7. Problem solving skills 8. Other (specify) |  |
| **Relationship between AFROHUN-Uganda One Health training activities and Employment** | | | |
| 228 | Employment (Select all responses that are true) | \| **Question** \| **Response** \| \| --- \| --- \| \| I was employed by the time I participated in AFROHUN-Uganda One health activities \| 1. Yes 2. No \| \| I continued on the same job after AFROHUN-Uganda One health activities \| 1. Yes 2. No \| \| I got a job after completing the AFROHUN-Uganda One health activities \| 1. Yes 2. No \| \| I am employed in a managerial position in my organization \| 1. Yes 2. No \| \| I was not employed by the time I participated in AFROHUN-Uganda One health activities \| 1. Yes 2. No \| \| I am not gainfully employed after participating in AFROHUN-Uganda One health activities \| 1. Yes 2. No \| | **Comments** |
| 229 | To what extent do you use the knowledge and skills acquired during AFROHUN-Uganda One health activities in your current job? | 1. Not at all 2. Limited Extent 3. Some extent 4. High extent 5. Very high extent |  |
| 230 | How would you characterize the relationship between AFROHUN-Uganda One health activities and your current job? | 1. AFROHUN-Uganda One health activities are by far the best in relation to my current job 2. Other fields of study could prepare me for this job as well 3. Another field of study would have been more useful for this job 4. The field of study does not matter very much for this job 5. Other (Specify) |  |
| 230 | \| **Please rate the extent to which you had acquired the following competencies and attributes at the time you participated in** AFROHUN-Uganda One health activities**.** Use the Likert scale to rate each of the aspects below:  **1. Not at all 2. Limited Extent 3. Some Extent 4. High Extent 5. Very High Extent** \| \| \| \| \| \| \| --- \| --- \| --- \| --- \| --- \| --- \| \| **Competences** \| **Not at all** \| **Limited extent** \| **Some extent** \| **High extent** \| **Very high extent** \| \| Field-specific theoretical knowledge \|  \|  \|  \|  \|  \| \| Field-specific practical knowledge/skills \|  \|  \|  \|  \|  \| \| Attitudinal change and perception to work \|  \|  \|  \|  \|  \| \| Critical thinking skills \|  \|  \|  \|  \|  \| \| Creativity \|  \|  \|  \|  \|  \| \| Collaboration \|  \|  \|  \|  \|  \| \| Communication skills \|  \|  \|  \|  \|  \| \| Leadership skills \|  \|  \|  \|  \|  \| \| Social (Influencing) skills \|  \|  \|  \|  \|  \| \| ICT skills \|  \|  \|  \|  \|  \| \| Analytical skills \|  \|  \|  \|  \|  \| \| Problem solving ability \|  \|  \|  \|  \|  \| \| Ability to take initiative \|  \|  \|  \|  \|  \| \| Entrepreneurial skills \|  \|  \|  \|  \|  \| \| Team building/working skills \|  \|  \|  \|  \|  \| \| People management skills \|  \|  \|  \|  \|  \| \| Customer orientation skills \|  \|  \|  \|  \|  \| \| Assertiveness, decisiveness, persistence \|  \|  \|  \|  \|  \| \| Accuracy, attention to detail \|  \|  \|  \|  \|  \| \| Planning, coordinating and organizing \|  \|  \|  \|  \|  \| \| Loyalty and integrity \|  \|  \|  \|  \|  \| \| Getting personally involved \|  \|  \|  \|  \|  \| \| Adaptability \|  \|  \|  \|  \|  \| \| Work ethics and integrity \|  \|  \|  \|  \|  \| \| Providing professional solutions to workplace challenges \|  \|  \|  \|  \|  \| | | |
| 231 | \| **Please state the extent to which the following attributes and competences are required in your current work.** Use the Likert scale to rate each of the aspects below:  **1. Not at all 2. Limited Extent 3. Some Extent 4. High Extent 5. Very High Extent** \| \| \| \| \| \| \| --- \| --- \| --- \| --- \| --- \| --- \| \| **Competences** \| **Not at all** \| **Limited extent** \| **Some extent** \| **High extent** \| **Very high extent** \| \| Field-specific theoretical knowledge \|  \|  \|  \|  \|  \| \| Field-specific practical knowledge/skills \|  \|  \|  \|  \|  \| \| Attitudinal change and perception to work \|  \|  \|  \|  \|  \| \| Critical thinking skills \|  \|  \|  \|  \|  \| \| Creativity \|  \|  \|  \|  \|  \| \| Collaboration \|  \|  \|  \|  \|  \| \| Communication skills \|  \|  \|  \|  \|  \| \| Leadership skills \|  \|  \|  \|  \|  \| \| Social (Influencing) skills \|  \|  \|  \|  \|  \| \| ICT skills \|  \|  \|  \|  \|  \| \| Analytical skills \|  \|  \|  \|  \|  \| \| Problem solving ability \|  \|  \|  \|  \|  \| \| Ability to take initiative \|  \|  \|  \|  \|  \| \| Entrepreneurial skills \|  \|  \|  \|  \|  \| \| Team building/working skills \|  \|  \|  \|  \|  \| \| People management skills \|  \|  \|  \|  \|  \| \| Customer orientation skills \|  \|  \|  \|  \|  \| \| Assertiveness, decisiveness, persistence \|  \|  \|  \|  \|  \| \| Accuracy, attention to detail \|  \|  \|  \|  \|  \| \| Planning, coordinating and organizing \|  \|  \|  \|  \|  \| \| Loyalty and integrity \|  \|  \|  \|  \|  \| \| Getting personally involved \|  \|  \|  \|  \|  \| \| Adaptability \|  \|  \|  \|  \|  \| \| Work ethics and integrity \|  \|  \|  \|  \|  \| \| Providing professional solutions to workplace challenges \|  \|  \|  \|  \|  \| | | |
| 231 | What were the major strengths of the **AFROHUN-Uganda One Health training activities** that are relevant to the job for which you were trained? | | |
