# Supplementary material for: Employment status of AFROHUN-Uganda one health alumni, and facilitators and barriers to application of the one health approach: a tracer study
Source: BMC Health Serv Res. 2022 Sep 27;22:1205. doi: 10.1186/s12913-022-08537-7 (PMC9513298; doi:10.1186/s12913-022-08537-7)
Supplement: Supplementary file 2 — Additional file 2. [file 12913_2022_8537_MOESM2_ESM.docx]

# **Qualitative interview guide to understand facilitators and barriers to incorporation of the OH approach**

1. Tell me about the work that your organisation does? Probe for activities that require a multidisciplinary approach, team work and collaboration. Also probe for: The core values and mission of the organisation? What activities do you do to achieve these core values?
2. What challenges do you often work towards solving in the communities you work?

Consider all problems – (for interviewer: HIV, public health, Environmental?

Probe: Do you consider these to require a multidisciplinary approach? If yes, how do you ensure a multidisciplinary approach to solving challenges in your area of work? If No, why not? (Could these challenges be addressed in a better way or differently and explain how?)

**Facilitators**

1. What makes it possible for your organisation to apply a multidisciplinary approach in solving day to day programmatic challenges? *(Keep in mind that these challenges include the community-based challenges requiring an intervention)*
2. Does your organisation culture support a multi-disciplinary approach to solving day to day challenges? If yes how? If yes, what conditions make it possible for your organisation to support a multi-disciplinary approach to solving day to day challenges?

Probe: Probe if employees work as a team, community engagement how they used OH approach

1. Do you have any human resource polices in place? If yes, how do human resource policies in this organisation affect a multi-disciplinary approach to solving day to day programmatic challenges?
2. Is task sharing done in this organisation? How is it done? Does it depend on some one’s expertise?
3. Have you ever heard of the one health approach? If yes, what do you know about the one health approach? Has your organisation ever hosted one health fellows? What was their impact on your day today activities?
4. Are there any steps your organisation is taking to promote the one health approach in the day today organisational activities? If yes, what steps are you undertaking? If No, why

**Barriers**

1. What makes it difficult for your organisation to promote a multidisciplinary approach to solving health challenges? *(Keep in mind that these challenges include the community-based challenges requiring an intervention)*
2. Does your organisation culture support a multi-disciplinary approach to solving day to day challenges? If No, why not? What are the conditions that make it difficult for your organisation to support a multi-disciplinary approach to solving day to day challenges? (Probe for nature of organisation e.g. private vs public, duration of existence, nature of work done, expertise in the organisation and policies).
3. What are the challenges you encounter in promoting a multi-disciplinary approach to solving day today challenges? How can these challenges be overcome?
4. How best can the one health approach be incorporated into organisations? Probe for: Incorporation in the private and public sector.

**Thank you for your time**
