# Supplementary material for: Employment status of AFROHUN-Uganda one health alumni, and facilitators and barriers to application of the one health approach: a tracer study
Source: BMC Health Serv Res. 2022 Sep 27;22:1205. doi: 10.1186/s12913-022-08537-7 (PMC9513298; doi:10.1186/s12913-022-08537-7)
Supplement: Supplementary file 3 — Additional file 3. [file 12913_2022_8537_MOESM3_ESM.docx]

Consolidated criteria for reporting qualitative studies (COREQ): 32-item checklist

Please indicate in which section each item has been reported in your manuscript. If you do not feel an item applies to your manuscript, please enter N/A.

For further information about the COREQ guidelines, please see Tong et al., 2017:

<https://doi.org/10.1093/intqhc/mzm042>

| **No.** | **Item** | **Description** | **Section #** |
| --- | --- | --- | --- |
| Domain 1: Research team and reflexivity | | | |
| Personal characteristics | | | |
| 1. | Interviewer/facilitator | Which author/s conducted the interview or  focus group? | Data were collected by TS and STW |
| 2. | Credentials | What were the researcher's credentials? E.g.  PhD, MD | TS-MPH  STW-Msc |
| 3. | Occupation | What was their occupation at the time of the  study? | Both are research associates at Makerere University School of Public Health  TS- One Health Workforce assessment and tracking thematic lead, AFROHUN UG/MakSPH |
| 4. | Gender | Was the researcher male or female? | TS-Male  STW-Male |
| 5. | Experience and | What experience or training did the researcher  have? | Qualitative research methods for 5 years |
|  | training |  |  |
| Relationship with participants | | | |
| 6. | Relationship | Was a relationship established prior to study?  commencement? |  |
|  | established |  | TS supervised some of the participants during the one health field activities |
| 7. | Participant knowledge | What did the participants know about the  researcher? E.g. Personal goals, reasons for doing the research | They knew that the researchers were |
|  | of the interviewer |  | The researchers were conducting the study to generate evidence about the one health training programs |
| 8. | Interviewer | What characteristics were reported about the  interviewer/facilitator? E.g. Bias, assumptions, reasons and interests in the research topic |  |
|  | characteristics |  | The interests in the research topic i.e. aimed to generate evidence of barriers and facilitators of application of the one health approach were reported. |
| Domain 2: Study design | | | |
| Theoretical framework | | | |
| 9. | Methodological | What methodological orientation was stated to  underpin the study? E.g. grounded theory, discourse analysis, ethnography, phenomenology, content analysis | Thematic-content analysis. **Page 7** |
|  | orientation and theory |  |  |
| Participant selection | | | |
| 10. | Sampling | How were participants selected? E.g. purposive,  convenience, consecutive, snowball | Purposive selection. **Page 7** |
| 11. | Method of approach | How were participants approached? E.g. face-  to-face, telephone, mail, email | Participants were approached via email, telephone and virtual platforms such as zoom and skype. **Page 7** |
| 12. | Sample size | How many participants were in the study? | A total of 12 respondents were interviewed. **Pages 2 and 7** |
| 13. | Non-participation | How many people refused to participate or  dropped out? What were the reasons for this? | 3 respondents declined to participate. They mentioned that they were too busy to participate in the study |
| Setting | | | |
| 14. | Setting of data  collection | Where was the data collected? E.g. home, clinic,  workplace | Data were collected remotely, at the participant’s convenience. Some participants were at their homes while others were at their workplaces. |
| 15. | Presence of non-  participants | Was anyone else present besides the  participants and researchers? | No |

| 16. | Description of sample | What are the important characteristics of the  sample? E.g. demographic data, date | Education level of the participants. All the participants had a minimum of a bachelor’s degree obtained at either Makerere University or Mbarara University of Science and Technology. |
| --- | --- | --- | --- |
| Data collection | | | |
| 17. | Interview guide | Were questions, prompts, guides provided by  the authors? Was it pilot tested? | Yes. These guides were pilot tested. **Page 8** |
| 18. | Repeat interviews | Were repeat interviews carried out? If yes, how  many? | No |
| 19. | Audio/visual recording | Did the research use audio or visual recording?  to collect the data? | Yes, only audio recording was done during data collection. **Page 8.** |
| 20. | Field notes | Were field notes made during and/or after the  interview or focus group? |  |
| 21. | Duration | What was the duration of the interviews or  focus group? | The interviews lasted 30-45 minutes. **Page 8** |
| 22. | Data saturation | Was data saturation discussed? | Data saturation was discussed. **Page 26** |
| 23. | Transcripts returned | Were transcripts returned to participants for  comment and/or correction? | No |
| Domain 3: analysis and findings | | | |
| Data analysis | | | |
| 24. | Number of data coders | How many data coders coded the data? | Three data coders coded the data. TS, JBI and RKM |
| 25. | Description of the  coding tree | Did authors provide a description of the coding  tree? | Yes. **Page 15** |
| 26. | Derivation of themes | Were themes identified in advance or derived  from the data? | Themes were derived from the data. Page 15 |
| 27. | Software | What software, if applicable, was used to  manage the data? | All transcripts were coded using the NVivo 12 Pro. Pages 2 and 8 |
| 28. | Participant checking | Did participants provide feedback on the  findings? | No. Participants did not provide feedback on the findings |
| Reporting | | | |
| 29. | Quotations presented | Were participant quotations presented to  illustrate the themes / findings? Was each quotation identified? E.g. Participant number | Yes, participant quotations were provided to illustrate the themes and finding. **Pages 16-20** |
| 30. | Data and findings  consistent | Was there consistency between the data  presented and the findings? | Yes, there was consistence between the data presented and findings |
| 31. | Clarity of major  themes | Were major themes clearly presented in the  findings? | Yes, major themes are clearly presented in the findings. Pages 15-20 |
| 32. | Clarity of minor  themes | Is there a description of diverse cases or?  discussion of minor themes? | No minor themes were derived. |

When submitting your manuscript via the online submission form, please upload the completed checklist as a Figure/supplementary file.

If you would like this checklist to be included alongside your article, we ask that you upload the completed checklist to an online repository and include the guideline type, name of the repository, DOI and license in the Data availability section of your manuscript.

Developed from: Allison Tong, Peter Sainsbury, Jonathan Craig, Consolidated criteria for reporting qualitative research (COREQ): a 32-item checklist for interviews and focus groups, International Journal for Quality in Health Care, Volume 19, Issue 6, December 2007, Pages 349–357, <https://doi.org/10.1093/intqhc/mzm042>
